# Minimally Invasive Interventional Procedures for Metastatic Bone Disease: A Comprehensive Review

**DOI:** 10.3390/curroncol29060332

**Published:** 2022-06-07

**Authors:** Nicolas Papalexis, Anna Parmeggiani, Giuliano Peta, Paolo Spinnato, Marco Miceli, Giancarlo Facchini

**Affiliations:** Diagnostic and Interventional Radiology Unit, IRCCS Istituto Ortopedico Rizzoli, Via Pupilli 1, 40136 Bologna, Italy; nicolaspapalexis@gmail.com (N.P.); giuliano.peta@ior.it (G.P.); paolo.spinnato@ior.it (P.S.); marco.miceli@ior.it (M.M.); giancarlo.facchini@ior.it (G.F.)

**Keywords:** bone metastases, imaging, interventional radiology, ablation techniques, embolization, high-intensity focused ultrasound ablation

## Abstract

Metastases are the main type of malignancy involving bone, which is the third most frequent site of metastatic carcinoma, after lung and liver. Skeletal-related events such as intractable pain, spinal cord compression, and pathologic fractures pose a serious burden on patients’ quality of life. For this reason, mini-invasive treatments for the management of bone metastases were developed with the goal of pain relief and functional status improvement. These techniques include embolization, thermal ablation, electrochemotherapy, cementoplasty, and MRI-guided high-intensity focused ultrasound. In order to achieve durable pain palliation and disease control, mini-invasive procedures are combined with chemotherapy, radiation therapy, surgery, or analgesics. The purpose of this review is to summarize the recently published literature regarding interventional radiology procedures in the treatment of cancer patients with bone metastases, focusing on the efficacy, complications, local disease control and recurrence rate.

## 1. Introduction

Metastases are the most common malignancy involving bone, where the skeleton is the third most frequent site for metastatic carcinoma, after lung and liver [1,2]. Therapeutic options are often limited due to the low expected survival of the patients, with few indications for surgical intervention. Nevertheless, skeletal-related events such as intractable pain, spinal cord compression, and pathologic fractures affect patients’ quality of life [3,4]. The goal of the current treatments is pain relief and functional status improvement and limiting treatment-related complications [5,6,7,8]. The management of bone metastases starts with percutaneous tumor biopsy in order to characterize the tumor histotype and to perform immunohistochemical analysis, fundamental for molecular targeted therapies [9,10,11,12]. The purpose of this review is to summarize the recently published literature regarding interventional radiology procedures in the treatment of bone metastases, focusing on the efficacy, complications, recurrence rate, and local disease control. In particular, transarterial embolization, electrochemotherapy, radiofrequency ablation, cryoablation, microwave ablation, magnetic resonance-guided focused ultrasound surgery, and percutaneous cementoplasty are discussed.

To date, radiotherapy is considered the gold standard for palliation for uncomplicated metastatic bone pain, while orthopedic surgery is preferentially recommended for patients with pathological or impending fracture, since radiotherapy alone does not contribute to bone stability [6,13]. The limitations of radiotherapy include radio-resistance of some tumor histotypes and the risk of pathological fractures due to radiation damage to weight-bearing bone structures. Another limitation is the maximum amount of radiation dose for one body site [14,15]. Orthopedic surgery in metastatic bone lesions has few indications as a first line treatment, in particular slow-growing tumors and patients with relatively good prognosis, where en-bloc resection may be considered a good option [16]. Surgery also has a primary role in the management of the acute instability of a vertebral body and spinal cord compression with neurological symptoms [13]. Novel minimally invasive treatments can both be an alternative for patients who did not benefit or are not eligible for conventional therapeutic options, or can be used in combination in order to provide the most efficient management for patients with bone metastases.

## 2. Embolization

Arterial embolization (AE) is an angiographic technique aimed to reduce the vascular supply of a bone lesion by selectively occluding its feeding arteries [17,18,19]. The injection of embolizing material is performed in order to only occlude the feeding vessels of the lesion, avoiding damage to the adjacent structures [20,21,22]. As mentioned above, skeletal-related events in patients with bone metastases can affect the quality of life. 

**Figure 1:**
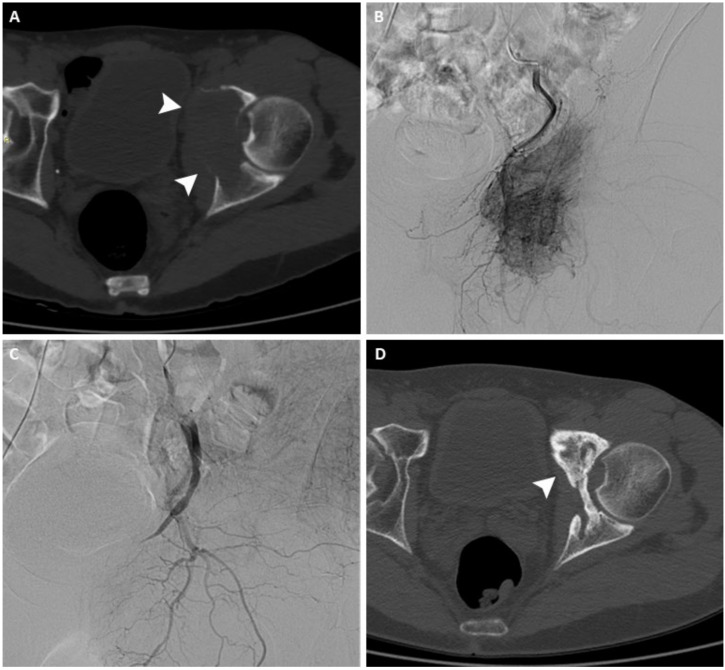
(**A**) Axial CT scan of the pelvis of a 63-year-old man with a painful left acetabular bone metastasis from kidney cancer (arrowheads). (**B**) Arteriography shows pathological vascularization originating from branches of the internal iliac artery. (**C**) After arterial embolization, arteriography demonstrates complete occlusion of the feeding vessels. (**D**) Axial CT scan performed 12 months after treatment shows signs of re-ossification and local disease control (arrowhead).

Several studies have reported the palliative effect of AE in patients who either did not benefit from radiotherapy or were not eligible for surgery, or both (Table 1) [23,24,25]. A study by Facchini et al., on 164 patients treated with palliative AE for metastases of the spine from variable primary cancers showed a reduction in the pain score and analgesic drugs consumption of over 50% in 97% of patients, with a mean pain relief duration of 9.2 months [26]. Similar results were previously reported by Rossi et al. in a study on 243 patients with bone metastases, with a reduction in pain score of 97% of patients and a mean duration of pain relief of 8.1 months [27]. A study on 18 patients with bone metastases [28] and one conducted on 39 patients with bone and soft tissue sarcomas [29] reported favorable outcomes when AE was performed with the addition of chemotherapeutic agents (transarterial chemo-embolization, TACE). In addition to pain reduction, AE has also proven to be effective for local disease control (Figure 1) [23,24,25,26,27]. In this regard, Facchini et al. reported a reduction in tumor size from a mean of 5.5 cm (range 3.5–7.5 cm) pre-embolization to a mean of 4.5 cm (range 3–5 cm) at the six-month follow-up [26]. 

In patients eligible for surgery, preoperative arterial embolization plays an important role in reducing intraoperative blood loss, improving tumor visualization and diminishing operative time [30,31,32,33,34]. Kato et al. conducted a study on 58 patients with renal and thyroid cancer bone metastases that received preoperative embolization to compare intraoperative blood loss when complete devascularization was achieved with incomplete devascularization. Intraoperative blood loss was lower with complete vs partial embolization (mean ± standard deviation, 809 ± 835 vs. 1210 ± 904 mL, *p* = 0.03); among patients with complete embolization, intraoperative blood loss was lower if the surgery was performed on the same day of the embolization [35]. Another recent study that included 41 patients with spinal and extra-spinal renal cell carcinoma metastases suggested that preoperative embolization was more effective in reducing blood loss when surgery was scheduled on the same day of the embolization [36]. Clausen et al. conducted a randomized controlled trial including 45 patients with metastases of the spine from variable primary cancers, 23 of whom received preoperative embolization and 22 surgery without preoperative embolization. Patients who received preoperative embolization had shorter operative times, but a reduction in blood loss was only statistically significant in patients with hypervascular metastases [37]. 

The majority of the available data supporting the importance of preoperative embolization involve the embolization of spinal metastases, with less data for extra-spinal bone metastases. A recent meta-analysis included seven studies reporting the results of preoperative embolization in metastases localized in long bones in terms of blood loss and blood replacement reduction. The level of evidence supporting the effectiveness of preoperative embolization in terms of blood loss and transfusion requirements was low, probably due to the retrospective nature of all studies and the small sample of patients, the lack of standardization of the embolization procedures, and the heterogeneity of the primary tumor type [38]. AE can be used as a complementary treatment to cementoplasty; a study conducted by Zhang et al., comparing the efficacy of arterial chemoembolization plus percutaneous cementoplasty versus cementoplasty alone for the treatment of pelvic bone metastases, reported a higher tumor response at one month for the combination treatment compared to cementoplasty alone [39]. 

The most reported adverse events are local skin discoloration or necrosis, post-embolization pain, embolization-related hemorrhage, and paresthesias [23,26,27,40]. Rossi et al. recorded minor embolization-related complications in 86/309 of procedures in patients treated for bone metastases of various anatomic locations, mostly post-embolization pain and paresthesia, and one major complication consisting of skin and subcutaneous tissue necrosis [27].

**Table 1:** Studies evaluating the role of transarterial embolization (TAE) in the management of bone metastases.

Main Author, Year	Reference	Study Design	PrO/Pa	Primary Tumor	Location of Metastasis	Included	Embolization	Control	Primary Outcome	Complications	Results
Wirbel, 2005	[17]	RET	PrO	Renal 45, other 17	Spine 41, pelvis 21	62	32	TAE vs. No TAE	Blood loss, blood replacement, operating time	2 m	Embolization reduces blood loss and need for blood replacement
Forauer, 2007	[25]	RET	Pa	Renal cell carcinoma	Pelvic 18, spine 5, other 16	21	39	0	Pain palliation	1 m, 2 M	Effective pain palliation was achieved in 36/39 sites, avg duration 5.5 months
Rossi, 2011	[27]	RET	Pa	Renal 84, lung 22, breast 20, other 117	Pelvis 154, spine 83, other 72	243	309	0	Pain palliation	86 m, 1 M	Effective pain palliation was achieved in 97% of procedures, avg duration 8.1 months
Robial, 2012	[34]	RET	PrO	Breast 28, lung 19, renal 16, other 30	Spine	93	35	TAE vs No TAE	Blood loss	ND	Embolization reduces blood loss and need for blood replacement
Kato, 2013	[35]	RET	PrO	Thyroid 39, renal 27	Spine	58	66	Optimal timing between embolization and surgery	Blood loss	0	Embolization reduces blood loss
Rossi, 2013	[40]	RET	Pa	Renal cell carcinoma	Pelvis 67, spine 32, other 8	107	163	0	Pain palliation	40 m, 1 M	Effective pain palliation was achieved in 96% of procedures, avg duration 10 months
Pazionis, 2014	[32]	RET	PrO	Renal cell carcinoma, thyroid carcinoma		118	53	TAE vs. No TAE	Blood loss, operating time, renal function impairment	2 m	Embolization reduces blood loss and need for blood replacement
Clausen, 2015	[37]	RET	PrO	Lung 17, Breast 8, Other 20	Spine	45	23	TAE vs. No TAE	Blood loss, blood replacement, surgery time	4 m, 1 M	Embolization reduces operative time; blood loss is reduced only in hypervascular metastases
Kim, 2015	[33]	RET	PrO	HCC	Femur 36, humerus 22, other 17	75	22	TAE vs. No TAE	Blood loss	ND	Embolization reduces blood loss
Facchini, 2016	[26]	RET	Pa	Renal 54, breast 22, other	Spine	164	178	0	Pain palliation	100 m, 1 M	Effective pain palliation achieved in 97% of procedures, avg duration 9.2 months
Jernigan, 2018	[19]	RET	PrO	Renal cell carcinoma	Femur	1285	135	TAE vs. No TAE	Transfusion requirements	ND	No effect on transfusion requirements
Çelebioğlu, 2021	[36]	RET	PrO	Renal cell carcinoma	Pelvis 12, spine 7, other 27	41	46	Optimal timing between embolization and surgery	Blood loss	15 m	Surgery should preferably be performed < 1 day after embolization

RET: retrospective, PRO: prospective; PrO: preoperative; Pa: palliative; m: minor; M: major. ND: not determined.

## 3. Electrochemotherapy

The functioning of electrochemotherapy (ECT) is based on the principle of reversible electroporation: it consists in a transient increase of cell membrane permeability to molecules, particularly chemotherapeutic drugs, that occurs when an electrical current is applied to the membrane [41,42]. In bone tumors, the treatment is performed by positioning 15-G needle electrodes inside the bone lesion under computed tomography (CT) or fluoroscopy guidance. Then, after bleomycin intravenous infusion, a pulsed electrical current is applied between the electrodes, causing the shift of the chemotherapeutic agent inside the targeted cells [43]. 

Mir et al., in 1991 were the first to use electrochemotherapy for the treatment of tumors, combining electroporation with the intravenous infusion of a chemotherapeutic drug [44]. Since then, the technique has been perfected, and it surged in 2006 when the European Standard Operating Procedures of ECT (ESOPE) were released, defining a standardization of the procedure for the treatment of superficial tumors for the first time [45], and finally updated in 2018, expanding the possible indications to larger and deeply-located tumors [46]. Preclinical studies have selected bleomycin and cisplatin as the most suitable chemotherapeutic agents for ECT, with an increased cytotoxicity of almost 8000 times for bleomycin and 80 times for cisplatin [47,48]. The first preclinical in vivo experience for the use of ECT in bone was performed in 2013 by Fini et al., who achieved good permeabilization with no negative effects on bone stability, mineralization, or osteogenic activity without signs of alterations on neurovascular adjacent structures [49].

In a clinical trial performed on 29 patients who received ECT for painful bone metastases, pain relief of more than 50% was achieved in 84% of patients at seven-month follow-up [50]. Another multicenter clinical study of 102 patients treated with ECT for bone metastases achieved an objective response to treatment by RECIST criteria in 40.4%, stable disease in 50.6%, and progression of disease in 9%, with a mean duration of follow-up of 5.9 ± 5.1 [51]. Out of 102 treated patients, 2 major and 11 minor complications occurred: one patient with advanced squamous cell carcinoma experienced local necrosis and one patient suffered a pathological fracture during the treatment; the minor complications consisted of persistent pain after the procedure that spontaneously resolved after a few weeks [51]. Gasbarrini et al. reported the use of minimally invasive ECT in one patient with melanoma metastasis in the body of L5; at the 48-month follow-up, the patient was pain-free with no progression of the disease [52]. 

## 4. Radiofrequency Ablation

Radiofrequency thermal ablation (RFA) of bone metastases is performed by percutaneous positioning of needle-electrodes into the lesion [53]. The needle-electrodes deliver a high-frequency electric current to the tumor, and cause protein denaturation and coagulation necrosis by frictional heating of the tip of the needle [54]. Ablating temperatures range from 70 to 90°; above 100°, tissue carbonization occurs, creating an isolating layer and reducing the volume of the ablation zone [55]. 

**Figure 2:**
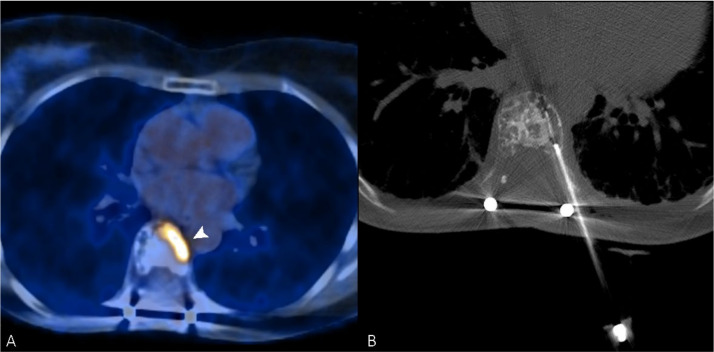
(**A**) PET/CT scan of a 56-year-old woman, which shows an intense uptake of 18F-FDG in correspondence with a painful vertebral metastasis from breast cancer in the body of T7 (arrowhead). (**B**) Radiofrequency ablation of the lesion performed through a transcostovertebral approach.

Among the various tumor ablation techniques, RFA is the most widely used and studied, not only in the treatment of liver, kidney, and lung tumors, but also in bone and soft-tissue lesions [55]. Since early 2000, percutaneous thermal ablation has been adopted in clinical practice as a palliative treatment for bone metastases (Figure 2) [56,57,58].

In 2002, Callstrom et al., described the use of RFA in 12 patients with painful metastasis, achieving a reduction in VAS pain score from 6.5 before treatment to 1.8 (*p* < 0.001) 4 weeks after treatment [56]. Gronemeyer et al. treated 10 patients with unresectable spinal metastases, achieving a 74% reduction in VAS score at the last follow-up, with a mean duration of follow-up of 5.8 months [57]. A retrospective multicenter study conducted on 128 metastatic lesions of the spine in 92 patients treated with RFA reported a reduction in VAS score from an average of 7.51 ± 2.46 pre-treatment to an average of 1.75 ± 2.62 at the six-month follow-up (*p* = 0.009) [58]. Dupuy at al. achieved an effective pain relief at the one-month and the three-month follow-up after performing RFA for bone metastases with an adverse event rate of 5%, mainly related to neurological damage [59]. 

For osteolytic metastases, RFA can be safely combined with cementoplasty, achieving pain palliation and bone stabilization; Zhao et al. reported the use of combined RFA and cementoplasty for metastases of the spine and long bones with excellent results on pain palliation and bone stabilization [60]. Levy et al. reported the use of RFA in combination with cementoplasty for the treatment of painful metastases of the spine with a reduction of the worst pain score from 8.2 ± 1.7 at baseline to 3.5 ± 3.2 at the six-month follow-up. Out of 100 treated patients, 4 adverse events occurred, and 2 resulted in hospitalization for pneumonia and respiratory failure [61]. 

## 5. Cryoablation

Cryoablation is a percutaneous thermal ablation where tumor tissue is cooled to extremely low temperatures by one or more probes filled with a compressed gas (usually argon) and placed within the lesion [62,63]. Exploiting the Joule–Thompson effect, as soon as the gas expands in the space surrounding the probe tip due to a rapid decompression, a temperature lower than −20 °C is achieved. The induced cooling damage consists of the formation of intracellular ice crystals that lead to cell destruction and to the impairment of vascularity based on endothelial damage, which compromises the blood supply, inducing local ischemia and devascularization [7,64,65]. Tumors close to large vessels are usually more difficult to treat, because during the ablation process, flowing blood conducts energy away from the target lesion (the so-called “cool sink” effect), impairing the achievement of an adequate cooling temperature at the edge of the metastasis [62,66]. The “cool sink” effect may cause local cancer recurrence due to inadequate treatment adjacent to major vascular structures [67,68]. Therefore, large hypervascular metastases should undergo percutaneous tumor embolization before the ablation session in order to achieve the best ablation outcome [69]. 

**Figure 3:**
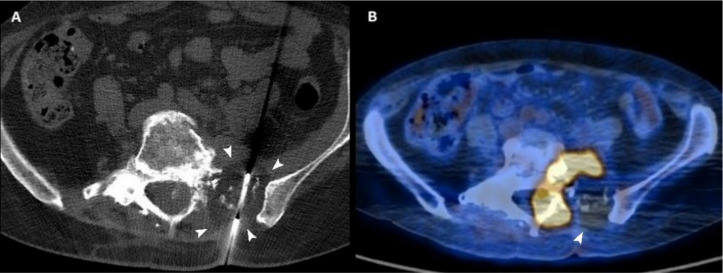
(**A**) Axial CT scan of a 54-year-old woman with a sacral metastasis from endometrial sarcoma treated with cryoablation for palliative intent. The ice ball is visible as a hypodense circle surrounding the tip of the needle (arrowheads). (**B**) 18F-FDG PET/CT scan performed 3 months after the procedure demonstrates the absence of pathologic radiotracer uptake in the ablated area (arrowhead).

In comparison with other thermal ablation techniques, during cryoablation it is possible to monitor the “ice ball” at the tip of the probe that can be directly visualized through CT imaging; since the margin of the ice ball indicates 0 °C, to assure complete tumor ablation the boundary of the ice ball should extend beyond the lesion itself (at least 5–8 mm) (Figure 3) [70,71,72]. 

Moreover, cryoablation is repeatable in cases of recurrent pain and offers the opportunity to use multiple cryoprobes simultaneously (up to 25), enabling the precise definition of the ablation zone through different probe placement geometries to match the shape of the target lesion and to create large ice balls (diameter > 8 cm), thus reducing the risk of possible residual disease [73]. Differently from other ablation techniques, cryoablation has proved to have an intrinsic analgesic effect, which implies less pain for the patient during and immediately after treatment [70,74,75]. However, CA is more expensive in comparison with other minimally invasive percutaneous treatments, even if it has been suggested it might be a potentially cost-effective alternative to radiotherapy (RT) for pain recurrence after RT in uncomplicated painful bone metastases [76,77]. 

In the management of bone metastasis, CA has proven to be an effective technique both for palliation purposes (pain ≥ 4 on a scale of 0–10) and for a curative aim in oligometastatic disease to ensure adequate local tumor control (LTC) [73,78,79,80,81,82,83]. Oligometastatic disease is defined as 1–5 metastases where all metastatic sites are considered safely treatable [84]. Even if there is still no univocal consensus in the literature, bone metastases with a size <2 cm and no cortical erosion have been associated with a better local tumor control after percutaneous image-guided CA or RFA [83,85]. 

Pain palliation is usually estimated through pain numeric rating scale administered before and after the procedure, self-assessment questionnaires on perceived quality of life, or evaluating the pre- and postprocedural analgesic requirements, while LTC is assessed through follow-up tumor imaging including CT, magnetic resonance imaging (MRI), or positron emission tomography (PET)/CT [86,87]. 

One of the consequences of bone necrosis induced by CA is the weakening of the bone structure, which predisposes the patient to delayed post-procedure fractures. For this reason, in patients affected by osteolytic metastatic disease at risk of fracture, especially in axial-loading sites as the periacetabular region or vertebral bodies, CA has successfully been associated with cementoplasty, which has demonstrated durable pain relief and stabilization [88,89,90,91,92]. Ferrer-Mileo et al. [78] conducted a systematic review of 22 studies regarding the use of cryoablation to control cancer pain, reporting a mean pain score decrease by 62.5% at 24 h post-procedure, 70% at 3 months, and 80.9% at 6 months. Moreover, opioid requirements decreased by 75% at 24 h and 61.7% at 3 months. Cryoablation has also been associated with a 44.2% improvement in quality of life after 4 weeks and 59.6% after 8 weeks. These results have been confirmed by many studies in the literature, proving that the CA of painful bone metastases induces a statistically significant improvement in patient pain level and perceived quality of life, and enables satisfactory local tumor control in oligometastatic disease (Table 2) [73,79,86,87,93,94,95,96,97]. 

Li et al. [98,99] evaluated the efficacy and safety of the combined regimen of cryoablation and zoledronic acid in 84 patients with painful bone metastases. Patients were randomly divided into three groups and underwent treatments of cryoablation plus zoledronic acid, cryoablation alone, and zoledronic acid alone. The results demonstrated that cryoablation plus zoledronic acid regimen induced a significant drop in the worst and average pain between week 1 and week 4 compared to zoledronic acid alone (*p* < 0.05), and a more durable effect on bone metastatic pain between week 12 and week 24 than cryoablation alone (*p* < 0.05), suggesting that a combined regimen was safe and more effective. Likewise, Di Staso et al. [100] used a propensity score-matching study design to compare radiotherapy (RT), cryoablation, or the combination of both in the treatment of patients with a solitary painful osseous metastasis. In a cohort of 175 patients, 25 underwent a radiation course 15 days after the cryoablation, 125 RT alone, and 25 cryoablation only. The results proved that 32% of patients of the cryoablation group and 72% of patients subjected to cryoablation followed by RT experienced a complete response compared with patients treated by RT alone (11.2%) in terms of pain relief, analgesics request, and self-rated quality of life. Therefore, the combination of RT and CA significantly improved the rate of complete response compared with cryoablation alone (*p* = 0.011). Cazzato et al. [69] recently assessed the safety, pain relief, and local tumor control achieved with percutaneous ablation with palliative or curative intent of 23 sacral bone metastases treated with RFA (9/23) or CA (14/23). Sixteen (70%) patients were treated with palliative and seven (30%) with curative intent. Within the given limits of the absence of a distinction among the type of ablative technique used, the numerical pain-rating scale at 32 months of follow-up was 2 ± 2 vs. 5 ± 1 at the baseline (*p* < 0.001) and 3/7 metastases (43%) treated for local tumor control showed progression during follow-up, suggesting that percutaneous ablation allows significant long-lasting pain control, but sub-optimal LTC. Similarly, Vaswani et al. [101] evaluated the effectiveness of RFA and CA in achieving local tumor control and pain palliation of sarcoma metastases in 64 patients, of which 13/64 with oligometastatic disease and 51/64 with widespread metastases. Thirty-one patients underwent CA, while 33 RFA and 27 ablated tumors were treated with adjunctive cementoplasty. In the group of oligometastatic disease, 3 of the original 13 ablated lesions were lost to follow-up, but the remaining treated lesions all exhibited local tumor control at follow-up imaging. The median pain scores decreased from 8 to 3 one month after the procedure (*p* < 0.001) and three patients reported increased pain after therapy (two treated with CA and one with RFA). A study by Zugaro et al. [102] evaluated pain relief improvement and quality of life in 50 patients with osteolytic solitary painful bone metastasis treated with CA (25 lesions) or RFA (25 lesions). Despite both techniques improved the self-rated quality of life (QoL), CA showed better results since 32% of patients experienced a complete response at 12 weeks (versus 20% of RFA) and the rate of complete response increased significantly with respect to baseline only in the group treated with CA. In both groups there was a significant change in the partial response with respect to baseline (36% in the CA group vs. 44% in the RFA group). The recurrence rate in the CA and RFA groups was 12% and 8%, respectively. The reduction in narcotic medication requirements with respect to baseline was only significant in the CA group (*p* = 0.0039). A large study by Auloge et al. [103] evaluated the complication rate and associated risk factors for bone tumor cryoablation in 239 patients who underwent cryoablation for a total of 320 primary or metastatic bone tumors. The total complication rate was 9.1% (29/320; 95% confidence interval (CI): 6%, 12.2%) and the major complication rate was 2.5% (8/320; 95% CI: 0.8%, 4.2%), where secondary fracture was the most frequent (1.2%). Minor complications included postprocedural pain, peripheral neuropathy, and temporary paresthesia. For all complications, the associated risk factors included long-bone cryoablation (odds ratio (OR), 17.8 (95% CI: 2.3, 136.3); *p* = 0.01), use of more than three cryoprobes (OR, 2.5 (95% CI: 1.0, 6.0); *p* = 0.04) and Eastern Cooperative Oncology Group performance status (ECOG-PS) greater than 2 (OR, 3.1 (95% CI: 3, 7.6); *p* = 0.01). For major complications, the associated risk factors were the use of more than three cryoprobes (OR, 23.6 (95% CI: 2.8, 199.0); *p* = 0.01) and age greater than 70 years (OR, 7.1 (95% CI: 1.6, 31.7); *p* = 0.01). In this regard, De Marini et al. [104] compared the safety profile of RFA and CA in the treatment of 367 bone metastases with and without a propensity score analysis, where 66 lesions underwent RFA and 301 CA. Major and minor complications were assessed according to the common terminology criteria for adverse events (CTCAE). There was no significant difference in the incidence of major complications between RFA (1/66; 1.5%) and CA (8/301; 2.7%; *p* < 0.001), while minor complications were more common with RFA than with CA (*p* < 0.001).

## 6. Microwave Ablation

Percutaneous microwave ablation (MWA) consists of the application of electromagnetic waves through an antenna placed within the tumor. The electromagnetic wave creates agitation of the water molecules, a process that generates heat and causes tumor coagulative necrosis [63,76,105]. 

In comparison with other percutaneous ablation techniques, MWA is less influenced by tissue impedance variability, including high impedance tissues such as bone or lung, and it is also less sensitive to the “heat sink” effect, which involves the dissipation of heat observed when a lesion is in close proximity to high flow blood vessels [7,63,106]. This enables higher intralesional temperatures, reducing the possible distortion of the ablation zone, and faster ablation time [63,71,107]. 

Although to date there are still few studies available, literature reports promising results regarding the palliative role of MWA for bone metastases and its efficacy in LTC, proving that MWA is a feasible and effective treatment for pain relief and quality of life improvement [107,108,109,110,111,112,113]. In a systematic literature review, Sagoo et al. [108] evaluated the use of MWA in the treatment of painful spinal metastases in eight studies, demonstrating MWA to be effective in achieving pain palliation for up to 6 months and local tumor control (success rate of 80-100%). Similarly, Cazzato et al. [109] conducted a systematic review regarding MWA safety and clinical efficacy and according to the seven studies analyzed, MWA is effective in achieving short-(1 month) and mid-term (4–6 months) pain relief after treating painful bone tumors, including skeletal metastases. The estimated pain reduction on the numerical rating scale for malignant lesions was 5.3/10 (95% CI 4.6–6.1) at 1 month and 5.3/10 (95% CI 4.3–6.3) at the last recorded follow-up (range 20–24 weeks in 4/5 studies). Aubry et al. [114] assessed the feasibility and efficacy of CT-guided MWA in the treatment of six osteolytic metastases, five osteoblastic metastases and five soft tissue sarcomas. At 1 month the percentage of necrosis estimated through follow-up imaging was 85 ± 30.4%, and the success rate was 80%. At 3, 6 and 12 months the success rate was 80%, 76.9% and 63.6%, respectively. At 12 months, four lesions (36.3 %) still had no recurrence. Recently, Yang et al. [115] evaluated the efficacy and safety of MWA in pain palliation of 18 bone metastases, demonstrating a significant pain reduction and morphine demand at 3 and 14 days after the procedure (6.83 ± 0.92 vs. 1.67 ± 0.97, *p* < 0.05 and 85.56 ± 17.23 vs. 32.78 ± 4.61, *p* < 0.05; 6.83 ± 0.92 vs. 0.94 ± 0.87, *p* < 0.05 and 85.56 ± 17.23 vs. 10.56 ± 8.73, *p* < 0.05, respectively).

Similarly to other percutaneous ablation techniques, MWA can increase the risk of bone pathological fracture, so it has been suggested to combine treatment with cementoplasty [111,112,116,117]. The MWA complication rate has been estimated at 4.0% (95% CI 1.9–7.3), where transient neural damage, skinburn, myofasciitis and local infection are the most common events [109,110]. In this regard, Kastler et al. evaluated the use of thermocouple probes for real-time temperature monitoring during bone MWA with the aim of preventing neural damage, which may occur when temperature reaches 45 °C [118,119]. According to the study, in a cohort of 16 patients, temperature was monitored during MWA procedure and did not increase over 43 °C; in eight cases MWA was interrupted because temperature reached 42 °C. No major complications occurred; minor complications included 5 cases of transient radicular pain. No side effects were noted in cases of proximity of the spinal cord to the tumor [118].

## 7. Magnetic Resonance-Guided Focused Ultrasound Surgery (MRgFUS)

MRgFUS bone lesion treatment consists of the use of a high-intensity focused ultrasound (HIFU) phased array system combined with an MRI system [71,120]. Differently from the aforementioned techniques, MRgFUS is a mini-invasive heat-based method, where a focused ultrasound beam generated by the transducer placed on the patient’s skin passes through the overlying tissues and reaches the target lesion [121]. The operating principle is twofold, and it is based both on the induction of thermal ablation and mechanical damage: the ultrasound beam energy is converted into thermal energy, where high-temperature exposure (65–85 °C with maximum acoustic energy of 2000 joules) induces tumor cell death through coagulative necrosis. It lasts for about 30 s, with a cool-down duration of 90 s between sonications [63,76,122]. The mechanical damage occurs with high intensity acoustic pulses, which generate high pressures and shear stress, potentially resulting in cell wall lysis [71,121]. 

MRI is pivotal in the pre-treatment phase in order to identify the bone lesion and at the same time to ensure that the trajectory of the ultrasound treatment beam does not hit adjacent organs or vascular and nervous structures. Moreover, MRI is fundamental as real-time imaging monitoring during the procedure and post-treatment to estimate the tumor response [71,121,123]. 

Pain palliation effect is likely due to local bone denervation, based on the degeneration of nociceptors and primary afferent sensory nerve fibers on the bone surface [124,125,126,127]. The effectiveness of MRgFUS as a mini-invasive treatment option for metastatic bone pain has been discussed in some literature reviews [71,120,128,129,130] reporting that more than approximately 70% of patients with radiation refractory metastatic bone pain experienced symptom improvement and a reduction of opioid usage after treatment. In addition, pain palliation has proved to be quickly achieved a few days after the procedure, lasting more than 3 months [63]. In particular, a systematic review and meta-analysis from Baal et al. [131] investigated the safety and efficacy of MRgFUS for painful bone metastases in 33 studies published between 2007 and 2019, for a total of 1082 patients. Complete response or partial response was 79% (95% CI 73–83%). The mean difference of pain scores between baseline and 1-month/3-month pain scores was −3.8 (95% CI: 4.3; −3.3) and −4.4 (95% CI: 5.0; −3.7), respectively. Similarly, a meta-analysis of 15 studies by Han et al. regarding the efficacy of MRgFUS in the treatment of patients with bone metastases achieved analog results, with a pain improvement compared with baseline of 2.54 at 0–1 week (95% CI: 1.92–3.16, *p* < 0.01), 3.56 at 1–5 weeks (95% CI: 3.11–4.02, *p* < 0.01), and 4.22 at 5–14 weeks (95% CI: 3.68–4.76, *p* < 0.01). 

A prospective study by Bongiovanni et al. [132] evaluated pain reduction in 12 patients with symptomatic bone metastases treated with MR-HIFU. Thirty days after the procedure, the results reported six (50.0%) complete responses to constant pain and six (50.0%) partial responses, with five (41.7%) and seven (58.3%) complete and partial responses to breakthrough cancer pain (BTCP), respectively. Morphine equivalent daily dose before treatment was 37.5 mg (range 0–270), while after treatment was 14.3 mg (range 0–270) and 7.3 mg (range 0–180) at 7 and 30 days, respectively. 

Despite the unquestionable advantage of being a repeatable radiation-free treatment, MRgFUS has some limitations. The lesion has to be accessible by the ultrasound beam without the juxtaposition of organs, vascular and nervous structures, non-targeted bone, or air, and the interface between the bone and tumor should be deeper than 10 mm from the skin surface [63,71,128,133]. Moreover, metastases located in the skull or in the spine cannot be treated, with the exception of the posterior elements below the level of the conus medullaris. MRgFUS treatment has a favorable safety profile, and the most common complications are skin burns, pain, vomiting, and delayed fractures [133,134,135]. High-grade and low-grade MRgFUS-related adverse events rates have been reported of 0.9% and 5.9%, respectively [131]. Moreover, MRgFUS has proved to be a cost-effective technique compared with medication-only approaches for the palliation of painful bone metastases in patients with medically refractory metastatic bone pain [136]. 

To date, the role of MRgFUS as a first-line treatment for painful bone metastasis has not been fully investigated. In this regard, Lee et al. [137] conducted a matched-pair study on 63 patients with bone metastases, where 21 were treated using MRgFUS and 42 RT. The results showed that both provided a similar overall treatment response rate, but MRgFUS was more efficient than RT in terms of faster pain relief and response duration (response rate at 1 week after treatment was 71% versus 26%, *p* = 0.0009, respectively). In comparison with RT, MRgFUS has the advantage of not only being ionizing radiation-free, but also of usually being effective after just a single treatment session [128].

## 8. Cementoplasty

Percutaneous cementoplasty (PC) is a minimally invasive technique consisting of the injection of cement, usually polymethylmethacrylate (PMMA), into a lytic bone lesion through a canula [138]. Differently from other interventional procedures aimed at tumor destruction, cementoplasty is a stabilization technique and it is used to consolidate the bone whose trabecular structure is weakened by a tumor, in order to reduce pain by the mechanical consolidation of fractured or pre-fractured bone [7]. Considering it has no effect on tumor growth progression, it can be performed alone with purely palliative intent, or as a complementary stabilization technique associated with other ablative treatments [139,140,141,142]. 

During the procedure, the PMMA polymerization phase is achieved through an exothermic reaction with temperature peaks of up to 75 °C which may play an accessory analgesic role through the destruction of nociceptors close to the lesion [76]. 

Since PMMA cement is resistant to compressive mechanical forces but susceptible to torsional forces, cementoplasty finds its main application in load-bearing bones as vertebral bodies and the acetabulum, while it is not recommended for lesions involving the diaphysis of long bones [7,76]. 

Currently, percutaneous vertebral augmentation procedures include vertebroplasty (VP) and balloon kyphoplasty (BKP), which unites the benefit of analgesia with the restoration of vertebral body height [143,144,145]. During kyphoplasty a balloon-like device is inflated inside the vertebral body, and its expansion restores vertebral body height, creating a cavity into which PMMA cement is then injected [143]. The access to the vertebral body can be transpedicular or extrapedicular, with a unilateral or bilateral approach. Following VP or BKP, vertebral stabilization is immediate and analgesic effect is obtained in a few days in about 90% of cases, with long-term palliation and improved mobility [146,147,148,149,150]. 

A systematic review regarding vertebral augmentation of cancer-related vertebral compression fractures in a total of 4235 patients [151] proved that both vertebroplasty and kyphoplasty significantly and rapidly reduce pain intensity, the need for opioid medication, and functional disabilities related to back and neck pain. A systematic review by Sadeghi-Naini et al. [152] compared the effects of VP and KP in patients with metastatic spinal lesion and both significantly improved pain, disability and health-related quality of life, even if no technique has proven to be substantially superior to the other. Similarly, Bae et al [153] retrospectively compared the outcomes of stabilization of painful metastatic fractures in 104 cancer patients subjected to BKP and 238 subjected to VP. The results demonstrated that an effective improvement in visual analog scale (VAS) score (≥3) was achieved in 206 patients (60%), but it was not significantly different between the two groups and BKP did not demonstrate significant pain improvement relative to VP. 

VP has been associated with higher incidence of asymptomatic and symptomatic cement leakage and with an increased risk of adjacent-level fractures in long-term follow-up [152,154,155]. Since progressive kyphosis due to vertebral compression fractures can lead to an increased loading of the spine anterior column with additional compression fractures, the restoration of vertebral body height with BKP has the beneficial effect of improving spinal sagittal balance [152]. BKP is burdened by the risk of iatrogenic damage to vertebral endplates with consequent cement leakage and it is approximately 2.5 times more expensive than vertebroplasty. VP should be preferred in cases of mild vertebral collapse or when the tumor involves the posterior somatic wall, since BKP could cause further tumor dissemination due to the balloon inflation [156]. 

Vertebral augmentation is contraindicated in patients with vertebral metastases causing neurological symptoms or osteoblastic metastases, acute infections, irreversible coagulopathies, and instability [7,146]. Complications include pain, infection, neuropathy and leakage of PMMA bone cement [157]. Cement leakage in the peri-vertebral veins, in the soft tissues or in the intervertebral discs occurs in approximately 70% of cases and is almost always asymptomatic [158]. Cement leakage in the spinal canal is rare, mostly occurring in lesions involving the posterior wall, and it can lead to spinal cord compression [159]. Intraforaminal leakage may be responsible for radiculopathy, which usually responds to nerve root blocks, surgical decompression or oral medications. Another complication is the development of venous cement embolism from the paravertebral veins [160]. 

Percutaneous cementoplasty has also proved to be a safe and effective choice for patients with painful osteolytic pelvic bone metastases, reducing pain and disability and improving function [161,162,163]. A study by Park et al. evaluated percutaneous cement injection in 178 patients with pelvic bone lesions achieving a pain reduction according to a numerical pain score from 6.1 to 2.4 (*p* < 0.01) and the maintenance of gait function in 68% of the patients [163]. 

As stated before, while percutaneous osteoplasty is widely used for the treatment of vertebral fractures, its application in metastatic long weight-bearing bones is still debated. In a literature review by Cazzato et al. [164] on 13 papers the use of percutaneous long bone cementoplasty (PLBC) in patients with bone metastases demonstrated a statistically significant pain improvement, with the development of a secondary fracture in 16/196 cases (8%, σ = 2.5). In 17% of cases PLBC was coupled to percutaneous bone stabilization, without any subsequent fracture. In this regard, the use of PC for metastases located in the proximal femur is still uncertain, because the anatomical site is associated with inadequate bone consolidation [165]. Deschamps et al. [166] retrospectively analyzed 21 patients who underwent cementoplasty for metastases of the proximal femur. The one-year pathologic fracture rate was 40.6% (7/21) and the risk of fracture was significantly higher for cortical involvement greater than 30 mm (7/11 vs. 0/10; *p* = 0.0005) and a history of a previous fracture of the lesser trochanter (3/3 vs. 4/18; *p* = 0.0009). A literature review by Kitridis et al. [167] on 12 studies compared the efficacy between augmented PC (APC) with fixation devices and PC for impending pathologic proximal femoral fractures from metastatic malignancy. For pain relief, results showed a mean difference in VAS score of −4.6 ± 1.7 for PC, and −4.3 ± 2.5 for APC (*p* = 0.41). Post-intervention fractures of the proximal femur occurred in 7% of patients with PC and in 5% of patients with APC (*p* = 0.4). The techniques did not show statistically significant differences, and both appeared effective in terms of palliation, prevention of pathologic fractures and weight-bearing recovery, but PC proved to be safer as no major complications were encountered after the procedure.

## 9. Technical Consideration

Interventional radiology procedures require careful evaluation of the patient’s functional status, blood tests, coagulation tests, and whenever contrast media is administered, kidney function. If local or systemic infections are suspected, the procedure should be rescheduled; any interventional radiology procedure, especially if bone-related, should be performed in a sterile setting to minimize the risk of infection. Prophylactic antimicrobial therapy can also be administered [140,168].

To date, there is no current evidence favoring the use of one ablation technique over another for the palliation of metastatic bone disease, as suggested by Gennaro et al. [169] in a literature review comparing the efficacy of some percutaneous thermal ablation techniques such as RFA, MWA, CA and MRgFUS in patients with painful bone metastases. The review included 11 papers (three on RFA, one on MWA, two on CA, and five on MRgFUS) for a total of 364 patients. Results reported a pain relief after 1 and 3 months up to 91% and 95% for all techniques, with a low incidence of minor and major complications.

The most appropriate approach should be chosen based on the features of the lesion to be treated with a possible combination of different procedures. The first condition to be evaluated is vascularization. As mentioned above, thermal ablation works by percutaneous delivery of extreme temperatures to the tumor causing cell death. The heat diffusion from the needle-electrode is influenced by the composition of the surrounding tissues and by the proximity of blood vessels for the risk of the heat sink effect [170,171]. Therefore, in the case of significant vascularization, embolization is recommended. Radiotherapy should be performed before arterial embolization, for its intrinsic mechanism of action based on the production of reactive oxygen species [172].

Another essential consideration is size. Larger volumes are more effectively treated with MWs or CA, or a combined approach can be planned to treat larger and more complex lesions [89,105,170]. RFA is appropriate for minor lesions (up to 3 cm). Sclerotic bone metastases are difficult to treat with RFA due to low thermal conductivity [170]. Microwave ablation, as mentioned above, is less affected by tissue composition, works faster, and can reach a larger ablation zone, up to 5 cm [7]. All of the percutaneous techniques may be performed under fluoroscopy or CT guidance, however, the main advantage offered by cryotherapy is the real-time visualization through CT scan of the ice ball [170]. Another potential interesting effect of mini-invasive treatments is intracellular antigen exposure to the immune system with activation of antigen-presenting cells (APCs), inducing an adaptive immune cancer response [173]. The main characteristics of the reported techniques are summarized in Table 3.

## 10. Conclusions

Currently, minimally invasive treatments represent a fundamental therapeutic option for patients with bone metastases. Arterial embolization determines the occlusion of the lesion-feeding vessels, providing pain palliation, local tumor control and reducing intraoperative blood loss. It can be used for the treatment of anatomical sites otherwise difficult to access, such as the spine or the pelvis. Moreover, it is safe and repeatable if needed, and it has demonstrated great efficacy in hypervascular lesions. Electrochemotherapy acts through the principle of reversible electroporation that increases the permeability of the tumor cell membrane to chemotherapeutic drugs. It is effective in pain palliation and local tumor control, and it can be safely used in the proximity of vascular or neural structures. RFA causes coagulative necrosis of the tumor through a high-frequency electric current. It is an accessible technique and provides predictable areas of ablation; however, it should be used for small lesions (up to 3 cm) and is not very effective in thick sclerotic bone. Cryoablation is based on the tumor cooling to extremely low temperatures, which induces cell necrosis. It is relatively safe for lesions located near vascular or neural structures due to the real-time visualization of the ablation site and it enables large ablation areas through different probe placement geometry. MWA causes tumor necrosis by electromagnetic waves that generate heat. It is associated with less predictable ablation areas compared to other techniques. MRgFUS is a non-invasive, radiation-free ablation procedure based on the use of a focused ultrasound beam that generates heat. It offers a real-time visualization of the ablation area, but it is less accessible compared to other ablation procedures. Moreover, it requires the optimal selection of the lesions, as it needs a good acoustic window and appropriate distance from vital structures. Cementoplasty offers bone stabilization through the injection of a cement polymer into the bone lesion, providing pain palliation and stability, but it has no effect on tumor progression control. 

Consequently, the choice of the most appropriate treatment for metastatic bone disease cannot be separated from a multidisciplinary evaluation that enables a tailored cancer therapy, with the aim of achieving optimal palliative effect and local tumor control, while ensuring the lowest risk of complications.

## Figures and Tables

**Table 2:** Studies evaluating the role of cryoablation in the management of bone metastases.

**Author,** **Year**	Reference	Study Design	Pa/LTC	Primary Tumor	Treatment Number	Follow-Up Duration	Complications	Results
Jennings, 2021	[79]	PRO	Pa	Various	66	6 months	3	Mean pain score improved from 22.61 points (95% CI: 23.45, 21.78) by 2 points at week 1 and reached clinically meaningful levels (more than a 2-point decrease) after week 8
Gallusser, 2019	[88]	RET	Pa, LTC	Various	18	12 months	1 delayed fracture	NRS score decreased significantly from 3.3 to 1.2 (*p* = 0.0024); LTC 63% (10/16)
Gardner, 2017	[94]	RET	LTC	Renal cell carcinoma	50	21.4 months	3 grade-3 and 1 grade-4, 5 delayed fractures	LTC 82% (41/50)
Arrigoni, 2022	[65]	RET	Pa, LTC	Various	28	3 months	1 grade-3	Mean VAS values dropped from 6.9 (SD: ± 1.3) to 3.5 (SD ± 2.6) (*p* < 0.0001); LTC 91% (10/11)
Coupal, 2017	[89]	RET	Pa	Various	48	2.25 months	none	Mean pain score decreased from 7.9 (range: 5–10) to 1.2 (range: 0–7) 24 h postintervention (*p* < 0.001)
Callstrom, 2013	[72]	PRO	Pa	Various	69	44 months	1 grade-3	Mean pain score decreased at 1, 4, 8, and 24 weeks from 7.1/10 to 5.1/10, 4.0/10, 3.6/10, and 1.4/10, respectively (*p* < 0.0001 for all)
Autrusseau, 2022	[95]	RET	LTC	Thyroid cancer	18	68 months	1 delayed fracture	Local tumor progression-free survivals at 1-, 2-, 3-, 4-, and 5-year was 93.3%, 84.6%, 76.9%, 75%, and 72.7%, respectively
McArthur, 2017	[96]	RET	Pa, LTC	Various	16	3 months	1 grade-1	Mean pain score improved for all patients (16/16), LTC 93.8% (15/16),
Hegg, 2014	[81]	RET	Pa, LTC	Various	12	5.7 months (Pa), 8.4 (LTC)	1 grade-2	Mean pain scores decreased from 7.0 ± 1.9 at baseline to 1.8 ± 1.2 (*p* = 0.00049), LTC 80%
Susa, 2016	[80]	PRO	LTC	Various	11	36 months	1 grade-1, 2 grade-2	2 patients developed local recurrence
Wallace, 2016	[86]	RET	Pa, LTC	Various	92 (10 in soft tissues)	6 months	2 grade-1, 2 grade-3	Decreased median pain scores were reported 1 day (6.0; *p* < 0.001, *n* = 62), 1 week (5.0; *p* < 0.001, *n* = 70), 1 month (5.0; *p* < 0.001, *n* = 63), and 3 months (4.5; *p* = 0.01, *n* = 28). LTC 90% (37/41) at 3 months, 86% (32/37) at 6 months, and 79% (26/33) at 12 months.
Tomasian, 2015	[87]	RET	Pa, LTC	Various	31	10 months	2 grade-1	NRS statistically significant decreased at 1 week, 1 month, and 3 months (*p* < 0.001 for all); LTC 96.7% (30/31)
McMenomy, 2013	[97]	RET	LTC	Various	52	21 months	2 grade-3	LTC 87% (45/52)

RET: retrospective, PRO: prospective; Pa: palliative, LTC: local tumor control, NRS: numeric rating scale.

**Table 3:** Features of the minimally invasive interventional procedures discussed.

Technique	Highlight	Advantage	Disadvantage	Anesthesia	Main Indication	Main Complications
Embolization	Endovascular occlusion of the arteries feeding the lesion	Fine visualization of the vascular supply of the lesion, capable of treating areas otherwise challenging to reach	Less effective if angiography shows poor vascularization of the lesion	Local	Highly vascular metastases in difficult to reach areas as pelvis or spine	Skin discoloration/necrosis, neural damage
Electrochemotherapy	Reversible electroporation with increased chemotherapeutic drug permeability	Safe near vascular and neural structures	Exposure to chemotherapeutic drugs	General	Large lesions in delicate locations	Local necrosis, pathological fractures
RFA	Application of high-frequency electric current to the lesion through needle-probes	Cost-effective, predictable areas of ablation	Small size of ablation area, risk of “heat sink” effect, not very effective in thick sclerotic lesions	Regional/sedation	Small lesions <3 cm	Damage to adjacent structures, more often neural
Cryoablation	Tumor tissue is cooled to extremely low temperatures by cryoprobes filled by a compressed gas	Very large ablation areas with complex geometries, real-time visualization of the ice ball	Costly, risk of “cool sink” effect	General	Large lesions, near vascular or neural structures	Post-procedural pain, neuropathy, fracture, skin burn
Microwave	Electromagnetic waves produced by an antenna that generates heat	Allows for large areas of ablation, do not suffer too much from heat sink effect	Less predictable ablation areas	Regional/sedation	Medium/large lesions not too close to neural or vascular structures	Transient neural damage, skin burn, fracture
MRgFUS	A focused ultrasound beam passes through the overlying tissues and reaches the target lesion	Non-invasive, radiation-free, real-time visualization of the ablation area	Costly, effective for lesions with the proper acoustic window and distant from vital structures	General	Deeply located lesions challenging to access, must have a good acoustic window	Skin burn, fractures
Cementoplasty	Cement polymer injection into a bone lesion	Bone stabilization, complementary to other ablation techniques	No effect on tumor growth control	Regional/sedation	Load-bearing bone asvertebral bodies and the acetabulum	Post-procedural pain, infection, neuropathy and leakage of bone cement
